# TRPV Channels in Mast Cells as a Target for Low-Level-Laser Therapy

**DOI:** 10.3390/cells3030662

**Published:** 2014-06-26

**Authors:** Lina Wang, Di Zhang, Wolfgang Schwarz

**Affiliations:** 1 Shanghai University of Traditional Chinese Medicine and Shanghai Research Center for Acupuncture and Meridians, Shanghai 201203, China; E-Mail: linawang1103@163.com; 2 Department of Mechanics and Engineering Science, Fudan University Shanghai, Shanghai 201203, China; E-Mail: dizhang@fudan.edu.cn; 3 Institute for Biophysics, Goethe-University Frankfurt am Main, 60438 Frankfurt, Germany; E-Mail: schwarz@biophysik.org

**Keywords:** laser light, TRPV activation, mast-cell degranulation, ATP, Ca^2+^, histamine

## Abstract

Low-level laser irradiation in the visible as well as infrared range is applied to skin for treatment of various diseases. Here we summarize and discuss effects of laser irradiation on mast cells that leads to degranulation of the cells. This process may contribute to initial steps in the final medical effects. We suggest that activation of TRPV channels in the mast cells forms a basis for the underlying mechanisms and that released ATP and histamine may be putative mediators for therapeutic effects.

## 1. Introduction

Various physical stimuli have been applied to the body surface for medical treatment. These treatments also include low-level laser therapy (LLLT) that uses irradiation in the mW/cm^2^ range of visible and near-infrared (NIR) light of 600 to 1000 nm (see, e.g., [1]). In this range of wavelengths, absorption within the human skin is very low [2,3,4], and the light can penetrate the tissue in the millimeter range [5]. Heat production by absorption is assumed to play a marginal role. At an output power in the mW range power densities of 1–5000 mW/cm^2^ were reported to be effective in modulation of inflammation [2,4,6]. Also for wound healing, in treatment of osteo- and rheumatoid arthritis and pain relief LLLT is applied (see, e.g., [4,7,8,9]. In particular red laser light had been introduced into Chinese medicine as laser acupuncture (see, e.g., [10]). Acupuncture points are characterized by a high density of mast cells, and stimulation by conventional needling leads to mast-cell degranulation, which seems to be an essential initial step in acupuncture-induced analgesia [11]. The mast-cell degranulation in acupuncture points cannot only be induced by mechanical stress, but also by high temperature which is used in Chinese medicine during application of moxibustion [12]. These results of the combined effects of mechanical stress and high temperature suggest the involvement of activation of TRPV channels that respond to both of these stimuli [13,14]. In fact, mast cells express TRPV1, TRPV2 and TRPV4 [15], and we asked the question whether these channels in general may form a target for LLLT.

## 2. Results and Discussion

In the following we will review and discuss effects of laser irradiation in the visible, infra-red (IR) as well as the ultra-violet (UV) range on ion channels of the TRPV family; effects of NIR have not been investigated. Since several of the medical effects, including wound healing [16] and anti-inflammatory effects [17], involve mast-cell activation, we will also consider to which extend modulation of TRPV channels are involved in this process. For the human mast-cell line HMC-1 expression of TRPV1, 2 and 4 was demonstrated [15]. We will, therefore, review the effects of laser light on these isoforms of TRPV, and discuss the role of the functional modulation in mast-cell degranulation.

### 2.1. Effects of Visible Light on TRPV

#### 2.1.1. TRPV1 as Target for Irradiation

We had previously investigated the effect of laser irradiation on TRPV1 [18]. To avoid interference with laser-induced effects on other ion channels of the TRPV family, we used the model system *Xenopus* oocyte with heterologously expressed TRPV1. The TRPV1-mediated current exhibited in solution with 2 mM Ca^2+^ a reversal potential of about −30 mV; a similar value of −20 mV has been reported for oocytes with solution containing 0.5 mM Ca^2+^ [19].

**Figure 1:**
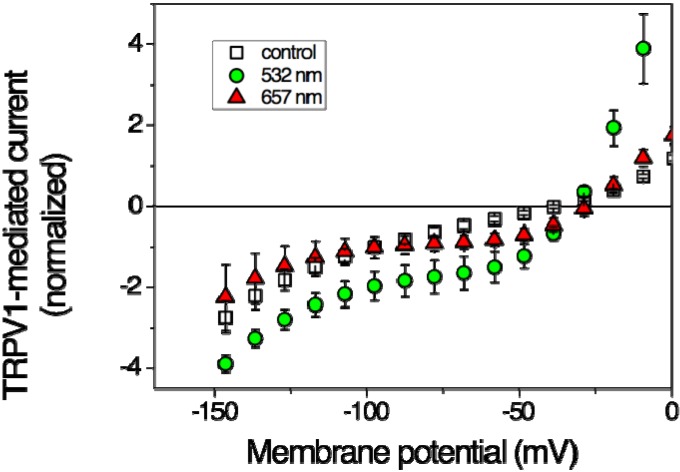
Effects of laser light on steady-state current-voltage curves of TRPV1-mediated current in *Xenopus* oocytes. Open squares represent data in the absence of irradiation (control), filled symbols at the end of a 2-min-lasting irradiation period of 637 nm (triangles up, 36 mW) and of 532 nm (circles, 40 mW), respectively at a power density of <500 mW/cm^2^. Data are averages of 3–5 oocytes ± SEM. (Based on data from Gu *et al.* 2012 [18]).

Figure 1 illustrates that TRPV1 can be activated by red laser light. With an output power of 36 mW already after 2-min irradiation a significant current increase could be detected, though in an only limited potential range; at −60 mV the current increased, nevertheless, by a factor of more than 2 (Table 1). The potential-dependent stimulation may indicate modulation of the voltage-dependent gating mechanism. Also, the effects of green laser light were investigated in the oocyte experiments [18]. Irradiation with green light of 532 nm and 40 mW output power produced a strong effect that reached steady-state after 2 min of irradiation over the entire potential range (Figure 1). With an increase by a factor of nearly 5 (at −60 mV) the effect was even more pronounced than that produced by the red light (Table 1).

**Table 1:** Effects of laser irradiation. Numbers in brackets refer to the respective publications, * is from unpublished work (A. Kutschireiter); **+** stands for stimulation, **−** for attenuation, n.a. for no data available.

	Red-Laser Irradiation	Green-Laser Irradiation	IR-Laser Irratiation	UV-Laser Irradiation
Wavelength	650 nm	532 nm	1.8–2.8 µm	≈300 nm
Power density (mW/cm^2^)	≤500	≤500	<3 × 10^6^ [20] <7 × 10^3^ [21]	15 × 10^3^ [22] 0.3 [23]
TRPV1 (current increase at −60 mV)	2.6 ± 0.5 [18]	4.7 ± 0.2 [18]	**−** heat avided [20]	**+** [23]
TRPV2 (current increase at −60 mV)	1.9 ± 0.5 [15]	n.a.	n.a.	n.a.
TRPV4 (current increase at −60 mV)	n.a.	≈3.7 *	**+** heat induced [21] **−** heat avided [20]	**+** [22]

#### 2.1.2. TRPV2 as Target for Irradiation

**Figure 2:**
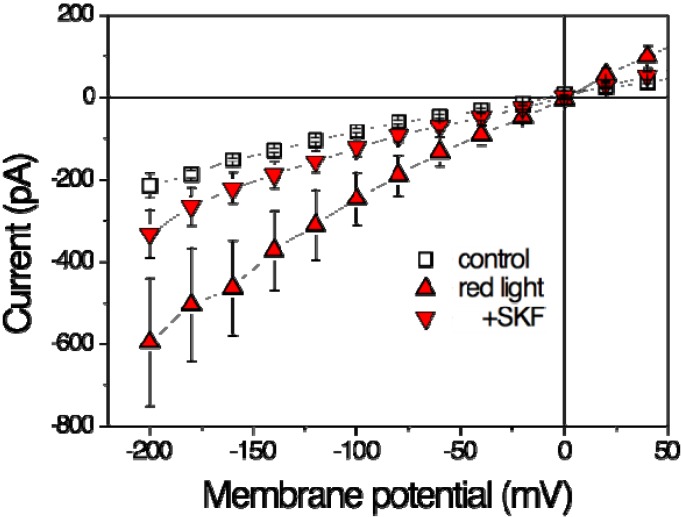
Effects of red laser light on steady-state current-voltage curves in HMC-1 cells (Human Mast Cell line). Whole-cell patch-clamp recording. Open squares represent data in the absence of irradiation (control), filled symbols at the end of a 20-min-lasting irradiation period of 640 nm (48 mW, ~500 mW/cm^2^) in the absence (triangles up, red light) and presence of 20 µM SKF96365 (triangle down, +SKF). Data are averages of five measurements ± SEM. (Based on data from Zhang *et al.* 2012 [15]).

Zhang *et al.* [15] investigate to what extent TRPV2 could contribute to mast-cell degranulation in response to physical stimuli including laser-light irradiation. Mast cells of the human cell line HMC-1 were exposed to red-laser irradiation of 640 nm with an output power 48 mW [15]. Patch-clamp recordings of steady-state current-voltage dependencies revealed a significant increase (Figure 2) in membrane current during 20 min of irradiation by a factor of about 2 (Table 1).

This current stimulation can be completely blocked by the TRPV inhibitor Ruthenium Red (RuR) [15]. Among the TRPV family, SKF96365 is specific for TRPV2 and can block most, but not all of the current suggesting that the laser-induced increase can be attributed to a large extent to activation of TRPV2 (Figure 2). Nevertheless, there seems to exist another RuR-sensitive current that can be stimulated by red laser light. Above, we have illustrated that at least the current mediated by TRPV1 is also stimulated by red laser light.

#### 2.1.3. TRPV4 as Target for Irradiation

Blue laser (405 nm) or green laser (532 nm) irradiation at about 150 mW/cm^2^ induced histamine release from rat basophilic leukemia (RBL-2H3) cells [24]. The non-specific inhibitor RuR for TRPV channels significantly blocked the irradiation-induced histamine release. Furthermore, immunocytochemical staining of TRPV4 increased after laser irradiation. The authors, therefore, conclude that TRPV4 stimulation was involved in the process of histamine release.

In *Xenopus* oocytes activation of TRPV4-mediated current by green laser light could be confirmed (A. Kutschireiter, unpublished); in orientating experiments with green laser light (532 nm, 40 mW, about 500 mW/cm^2^) a pronounced stimulation to a constant value within less than a minute was observed (Table 1).

### 2.2. Effects of IR Light on TRPV

In contrast to visible red light, IR light is absorbed by the tissue and converted to heat. Since the ion channels of the TRPV family are also sensors for high temperature [25], it can be expected that IR light activates TRPV channels. The effect of heat on mast-cell degranulation had been investigated [15], and it could be demonstrated that indeed heat application resulted in degranulation associated with increase intracellular Ca^2+^ and ATP release [26] (see Section 2.5). In addition, IR laser light (1.875 µm, 320 mW, 2.7 × 10^6^ mW/cm^2^, 20–60 × 10^3^ mJ/cm^2^) triggered TRPV4-mediated transient membrane potential variations in retinal ganglion cells of mice and in vestibular cells of rats [21]. An increase of the temperature by about 10 °C occurred during the irradiation and can account for TRPV4 stimulation [21].

Application of IR irradiation (2.780 µm at 260–6500 mW/cm^2^, 3.98–33.17 × 10^3^ mJ/cm^2^) could significantly relieve capsaicin- and hypotonicity-induced nociception [20]. Furthermore, capsaicin- or hypotonicity-induced Ca^2+^ influx in TRPV1 and TRPV4 over-expressing HeLa cells were attenuated by this laser irradiation. These results suggest that TRPV1 and TRPV4 are inhibited by IR irradiation [20]. In this study, water was sprayed during irradiation to avoid skin burn, and the authors claimed no obvious temperature changes in their *in vitro* tests because of the perfusion of bath solution [20]. Therefore, the modulation of TRPV1 and TRPV4 by laser irradiation cannot be attributed to heating.

### 2.3. Effects of UV Light on TRPV

Solar ultraviolet (UV) irradiation is a major cause of premature aging of skin. Lee *et al.* [23] confirmed that UV irradiation (300–320 nm, 0.3 mW/cm^2^, 36 mJ/cm^2^) induced slow and persistent membrane current and Ca^2+^ influx in HaCaT cells, a human keratinocyte cell line. This current was inhibited by TRPV1 inhibitors (capsazepine and RuR). The UV-induced matrix metalloproteinase-1 (MMP-1) was decreased by TRPV1 inhibitors and was facilitated by capsaicin. Knock-down of TRPV1 using siRNA transfection also decreased MMP-1 expression as well as UV-induced Ca^2+^ influx in HaCaT cells. Another non-selective cation channel highly expressed in epithelial skin cell is TRPV4. Moore *et al.* [22] showed that Ca^2+^ influx into keratinocytes in response to UVB (295 nm, 15 × 10^3^ mW/cm^2^, 600 mJ/cm^2^) depends on TRPV4. External topical application of a TRPV4-selective inhibitor (GSK205) attenuates UVB-evoked nocifensive behaviour and endothelin-1 expression, and keratinocyte-derived TRPV4 was suggested as a therapeutic target for UVB-induced sunburn.

### 2.4. TRPV Modulation and Mast-Cell Activation

In the above sections we illustrated and discussed that laser irradiation in the visible as well as IR and UV range results in the activation of TRPV channels, in particular of TRPV1, 2 and 4 seemed to be effected.

The activation of membrane current by red laser light is accompanied by degranulation of the mast cells [15,18,27]. Patch-clamp experiments revealed that the degranulation becomes also apparent by an increase in membrane capacitance [15]. Nevertheless, the observed increase in membrane surface by about 40% cannot account for the increase in membrane current by a factor of 2 (see Table 1) indicating that the red laser light stimulates TRPV2 channel activity.

Green laser light stimulates TRPV1 when expressed in *Xenopus* oocytes [18], and activation of TRPV1 also leads to mast cell degranulation and could be demonstrated by direct activation of TRPV1 by the specific agonist capsaicin [18].

Effects of NIR, IR [28,29] and UV [30,31] light on mast cell activation have been reported but involvement of TRPV has not been investigated.

### 2.5. Effect of Laser Irradiation on Intracellular Ca^2+^ Activity and ATP Release Mobilisation

**Figure 3:**
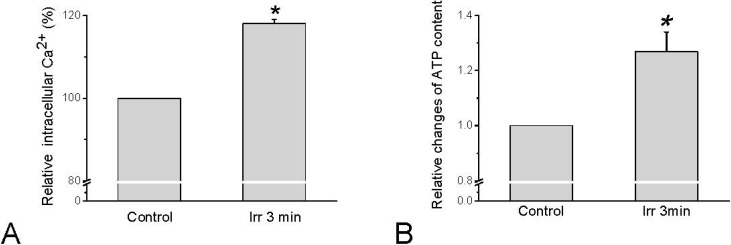
Changes in [Ca^2+^]_i_ and extracellular ATP in HMC-1 cells in response to 657 nm and 280 mW/cm^2^ laser irradiation for 3 min. (**A**) Quantitative analysis of several sets of cells normalized to basal [Ca^2+^]_i_-dependent fluorescence before irradiation (=100%). Data represent averages ± SEM (n = 15 cells, * *p* < 0.01 compared to control); (**B**) Relative changes of extracellular ATP averaged from all sample. The data represent averages ± SEM (n = 27, * *p* < 0.01 compared to control, and are based on Wang *et al.* 2013 [33]).

Since TRPV channels are permeable for Ca^2+^, Ca^2+^ influx can be expected to occur in response to TRPV channel activation. Concerning HMC-1 cells, intracellular calcium activity ([Ca^2+^]_i_) indeed increased in response to irradiation by red laser light of 657 nm (280 mW/cm^2^, 54 × 10^3^ mJ/cm^2^) (Figure 3A). Interestingly, this elevation of [Ca^2+^]_i_ was independent on extracellular Ca^2+^. With the same irradiation parameters that stimulate [Ca^2+^]_i_ increase an elevation of extracellular ATP could be detected (Figure 3B), and the [Ca^2+^]_i_ was demonstrated to trigger exocytotic release of ATP [32].

**Figure 4:**
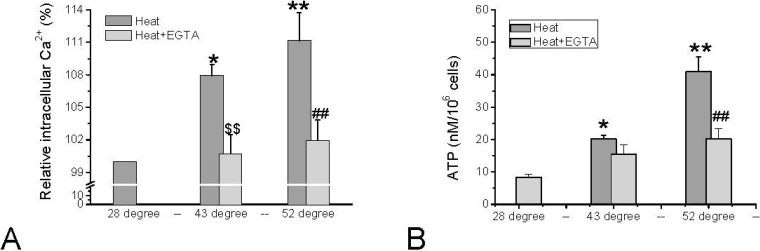
Changes in [Ca^2+^]_i_ and extracellular ATP in HMC-1 cells in response to noxious heat that activates TRPV1 (43 °C) and TRPV2 (52 °C), and either in the absence (dark grey bars) or presence (light grey bars) of 5 mM EGTA. (**A**) Quantitative analysis from several sets of experiments normalized to basal [Ca^2+^]i–dependent fluorescence at 28 °C (=100% of Calcium Green-1 loaded HMC-1 cells). Data represent averages ± SEM from N = 3–4 independent experiments (* *p < 0.0*5, ** *p* < 0.01, compared to 28 °C; $$ *p* < 0.01 compare to 43 °C, ## *p* < 0.01, compared to 52 °C); (**B**) ATP content in cell suspension after 3 min of incubation at the respective temperatures. Data represent averages ±SEM from n = 6–42 samples of N = 3–11 independent experiments (* *p* = < 0.05 and ** *p* < 0.0001, compared to control; ## *p* < 0.0001, compare to 52 °C). Data in (**A)** are unpublished, data in (**B**) are based on Hu *et al.* 2014 [26].

The thermal activation of TRPV1 and TRPV2 is characterized by threshold temperatures of 43 °C and 52 °C, respectively. These noxious temperatures resulted in TRPV1- and TRPV2-dependent influx of Ca^2+^ in HMC-1 leading to elevated [Ca^2+^]_i_ (Figure 4A). Only exceeding a temperature of 43 °C increased [Ca^2+^]_i_, and a further significant increase was observed when exceeding 52 °C. The relative changes in intracellular calcium activity were monitored by measuring the [Ca^2+^]_i_–dependent fluorescence of Calcium Green. The fluorescence signal of Calcium Green was reported to be temperature-dependent [34,35], but at the temperatures exceeding 28 °C the signal is hardly affected, and the changes illustrated in Figure 4A will not significantly be altered. As was illustrated for stimulation of mast cells by red laser light (see above), the temperature-induced elevation of [Ca^2+^]_i_ triggers release of ATP, which becomes apparent in elevated extracellular ATP content (Figure 4B).

The increase in [Ca^2+^]_i_ as well as in extracellular ATP induced by the noxious temperatures can partially be blocked by the Ca^2+^ chelator EGTA suggesting that influx of Ca^2+^ via TRPV1 and TRPV2 is involved (Figure 4B). In fact, inhibition of TRPV2 by 20 µM SKF96365 could partially abolish the increase of the extracellular ATP level induced by 52 °C by 27.9% ± 10.0% (N = 3 independent experiments (unpublished)).

### 2.6. Underlying Mechanisms for Laser-Light-Induced TRPV Modulation

**Figure 5:**
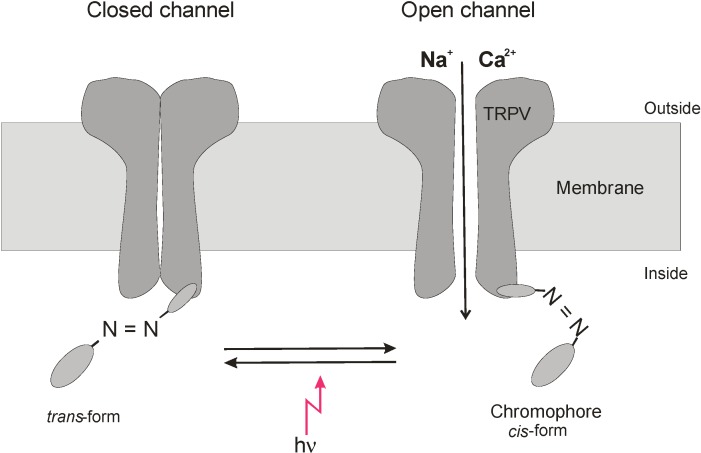
Modulation of TRPV channel gating by light-switched ligand. Putative modulation of an azo-chromophore between *cis*- and *trans*-form by light leading to activation of TRPV channel opening. As an example TRPV activation by the *cis*-form is cartooned.

Direct modulation of TRPV channels by absorption of visible and NIR light can hardly be expected, but the existence of various chromophores has been reported that may act as switches to modulate the function of membrane proteins [1,36,37,38,39,40,41]. Laser irradiation could induce the histamine release in RBL-2H3 mast cells dependent on TRPV4 activation [20], and its wavelength for activation of the histamine release was consistent with the absorption bands of cytochrome c oxidase [42]. Activation of cytochrome c oxidase has been demonstrated to be involved in light-induced stimulation of the Na, K-ATPase [36] and tissue healing [43,44]. The light-induced stimulation of TRPV ion channels we observed in our experiments developed within a few minutes (see, e.g., [18]); therefore, we consider the involvement of cytochrome c oxidase not as a dominating process. It could be demonstrated that light can modulate in channel activity of TRPs by isomerising photochromic antagonists between *trans* state and *cis* form [37]. In particular, azobenzenes were reported to act as photoswitches for a variety of biomolecules including membrane proteins [41]. The azo-benzene-derivative AC4 of the TRPV1 antagonist capsazepine, for example, exists at 440 nm in its *trans* and at 360 nm in its *cis* configuration, and depending on the stimulus for TRPV1 activation the *trans* or *cis* configuration can reversibly act as antagonist (comp. Figure 5). Also, other members of the TRP family are reported to be involved in phototransduction. For example, TRPA1 was demonstrated to be essential for extraocular pathway in human melanocytes when exposed to UV radiation. The photocurrent is mediated by G protein-coupled receptors and leads to rapid Ca^2+^ mobilization [38]; also, in this process, photosensitive agents are involved. TRPA1was also reported to be activated by UV light in HEK293 cells [39]. [Ca^2+^]_i_ as well as whole-cell currents and open probability of TRPA1 were potentiated by 350 nm UVA light at power density of 580 mW/cm^2^ and doses of 34.8–104.4 × 10^3^ mJ/cm^2^. It is interesting to note that photosensitising agents could shift light activation of TRPA1 to longer wavelengths of 490 nm and 590 nm [39]. Whether a light-switchable physiological chromophore can account for the observed stimulation of the ion channel activity of TRPV needs further investigation.

The possible mechanism for TRP gating may involve direct binding by lipids such as diacylglycerol and polyunsaturated fatty acids at specific sites on the channels proteins [40]. Single-channel currents could be evoked by light in rhabodomere-attached patches from intact isolated photoreceptors of *wt Drosophila*, while mutants lacking TRP and TRP-like proteins are devoid of light-inducible channel activity. For TRPV4, it could be demonstrated that activation by hypotonicity or heat requires interaction with phosphatidylinositol-biphosphate [45]; in general, ligand-dependent activation of thermo-sensitive TRP channels has been discussed [43].

The effects of irradiation we have summarized in this review and that led to activation of TRPVs and/or mast cell degranulation included results mainly obtained with laser irradiation (see also [36]), but also light from light-emitting diodes (e.g., [23]), filtered light or even broad-band irradiation (e.g., [30,31,38]). Since similar effects were observed, coherence of light seems not to be essential for the observed effects.

## 3. Conclusions

We have shown in this review that laser irradiation in the visible and IR as well as UV range can modulate the function and expression of TRPV ion channels, and in particular TRPV1, TRPV2, and TRPV4. This may form the basis for effect of LLLT. As Ca^2+^-permeable ion channels, their activation may contribute to the laser-induced increase in intracellular Ca^2+^ that triggers degranulation and endocytotic release of ATP. Such light-induced mechanism may contribute to the basis of the medical effects of LLLT. This hypothesis still needs confirmation in animal tests and clinical trials.
